# 32. DIGGING DEEPER IN THE PROTEOME OF SCHIZOPHRENIA

**DOI:** 10.1093/schbul/sby014.131

**Published:** 2018-04-01

**Authors:** Daniel Martins-De-Souza

**Affiliations:** University of Campinas (UNICAMP)

## Abstract

**Overall Abstract:** Advances in genomics and transcriptomics have yielded novel insights for the pathophysiology of schizophrenia, moving the field forward by providing new substrates for the development of treatment strategies. Interestingly, this has led to a large gap in knowledge in the field, as the impact of genomic variability or alterations in transcript expression is dependent on the next level of gene expression. While proteomics has lagged behind other disciplines in schizophrenia research, several groups are utilizing proteomics approaches to ask and answer the largest possible questions in translational schizophrenia research. Proteomics has evolved as a field very quickly, going beyond the characterization of expression levels of one or a few proteins. With precise quantification of protein expression and degradation, characterization of post-translational modifications as well as the detection of low abundant proteins as putative biomarkers, we will show several different state-of-the-art proteomics approaches applied to the schizophrenia substrate. James Meador-Woodruff (University of Alabama at Birmingham) will provide a brief overview of the field, and present new data showing abnormalities of lipid and carbohydrate modifications on receptor proteins in schizophrenia, as well as abnormal levels of key enzymes associated with these abnormal protein modifications. Robert McCullumsmith (University of Cincinnati, USA) will add pivotal information on the long-standing synaptic hypothesis of schizophrenia by depicting the PSD95 interactome in normal and schizophrenia brain, connecting the excitatory postsynaptic proteome to Big Data analytics. The other two speakers will show have translational proteomics data can be used to develop clinical biomarkers. Dr. Mariana Fioramonte will present how the use of the latest tools in proteomics can tell us more about brain proteomics and protein interactomics. Finally, David Cotter (Royal College of Surgeons, Ireland) will show that proteins associated to the complement system present in the blood plasma of adolescent subjects experiencing psychosis can be predictive broadly in the vulnerability of adult psychiatric disorders. This symposium provides relevant on the biological and biochemical aspects of schizophrenia and proposes potential applications that might be, after adjustments and validations, implemented in the clinic in the future, towards a personalized medicine concept.

